# Myopic Macular Hole and Detachment after Gene Therapy in Atypical RPE65 Retinal Dystrophy: A Case Report

**DOI:** 10.3390/genes15070879

**Published:** 2024-07-04

**Authors:** Fabrizio Giansanti, Cristina Nicolosi, Dario Giorgio, Andrea Sodi, Dario Pasquale Mucciolo, Laura Pavese, Liliana Pollazzi, Gianni Virgili, Giulio Vicini, Ilaria Passerini, Elisabetta Pelo, Vittoria Murro

**Affiliations:** 1 Eye Clinic, Neuromuscular and Sense Organs Department, Careggi University Hospital, 50134 Florence, Italy; fabrizio.giansanti@unifi.it (F.G.); andreasodi2@gmail.com (A.S.); dario.mucciolo@gmail.com (D.P.M.); pollazzil@aou-careggi.toscana.it (L.P.); gianni.virgili@unifi.it (G.V.); vittoria.murro@unifi.it (V.M.); 2 Department of Neurosciences, Psychology, Drug Research and Child Health (NEUROFARBA), University of Florence, 50121 Florence, Italy; dario.giorgio87@gmail.com (D.G.); laura.pavese@unifi.it (L.P.); giulio.vicini@gmail.com (G.V.); 3 Azienda USL Toscana Nordovest, 56121 Pisa, Italy; 4 SODc Diagnostica Genetica, Careggi University Hospital, 50134 Florence, Italy; ilariapasserini70@gmail.com (I.P.); peloe@aou-careggi.toscana.it (E.P.)

**Keywords:** retinitis pigmentosa, voretigene neparvovec, macular hole, RPE65, gene therapy, complications

## Abstract

Purpose: To report a case of macular hole and detachment occurring after the subretinal injection of Voretigene Neparvovec (VN) in a patient affected by atypical RPE65 retinal dystrophy with high myopia and its successful surgical management. Case description: We report a case of a 70-year-old man treated with VN in both eyes. The best corrected visual acuity (BCVA) was 0.7 LogMar in the right eye (RE) and 0.92 LogMar in the left eye (LE). Axial length was 29.60 mm in the RE and 30.28 mm in the LE. Both eyes were pseudophakic. In both eyes, fundus examination revealed high myopia, posterior staphyloma, and extended retinal atrophy areas at the posterior pole, circumscribing a central island of surviving retina. Both eyes were treated with VN subretinal injection, but a full-thickness macular hole and retinal detachment occurred in the LE three weeks after surgery. The patient underwent 23-gauge vitrectomy with internal limiting membrane (ILM) peeling and the inverted flap technique with sulfur hexafluoride (SF6) 20% tamponade. Postoperative follow-up showed that the macular hole was closed and the BCVA was maintained. Conclusions: Our experience suggests that patients with atypical RPE65 retinal dystrophy and high myopia undergoing VN subretinal injection require careful management to minimize the risk of macular hole and detachment occurrence and promptly detect and address these potential complications.

## 1. Introduction

Voretigene Neparvovec (VN; brand name Luxturna^®^, Spark Therapeutics Inc., Philadelphia, PA, USA) is the first in vivo gene therapy for the eye approved for the treatment of retinal degeneration in patients affected by inherited retinal dystrophy caused by biallelic mutations in the RPE65 gene. It is the first approved recombinant adeno-associated virus vector-based gene therapy designed to deliver a functioning copy of the human RPE65 gene into viable retinal pigmented epithelium cells via a single subretinal injection in order to potentially restore the visual cycle [1]. Despite several clinical trials showing the efficacy of the drug and a good safety profile [2,3], some complications have been reported so far. These complications include maculopathy, retinal tears, intraocular pressure rise, pseudopapilledema, cataracts, self-resolving subconjunctival and/or retinal hemorrhage, macular holes, and retinal tears. On the other hand, different adverse events such as subretinal deposits [4], inner retinal swelling [5], iatrogenic choroidal neovascularization [6], vitreitis [7], and chorioretinal atrophy [8] have only been described in real-world studies and not previously reported in pre-clinical studies.

Herein, we report the case of an RPE65 patient with uncommon phenotype expression and high myopia who underwent subretinal VN administration and developed a macular hole and, consequently, macular detachment. We also report the successful surgical management of the complication and our considerations of the possible concurrent risk factors for macular hole occurrence in this atypical RPE65 phenotype.

## 2. Case Report

This study adhered to the tenets of the Declaration of Helsinki. Written informed consent was obtained from the patient. 

A 70-year-old male patient affected by retinal dystrophy was referred to our center for inherited retinal dystrophies at Careggi University Hospital, Florence, Italy.

The patient was under treatment for systemic arterial hypertension and was affected by severe bilateral hearing loss, needing hearing aids since the age of fifty. He had no familiarity with eye diseases and was diagnosed with retinitis pigmentosa when he was three years old. The onset symptoms and signs were hemeralopia, vision loss, and visual field constriction.

A genetic test examination for inherited retinal dystrophies was performed after obtaining informed consent and pre-test genetic counseling. Ten mL of peripheral blood was obtained from the antecubital vein using EDTA-containing vials. DNA was extracted from 200 μL of peripheral blood by using the automated DNA extractor QIAsymphony SP workstation (QIAGEN GmbH, Germany), according to the manufacturer’s protocol. Genomic DNA from the patient was used for identifying mutations by a targeted next-generation sequencing (NGS) gene panel for inherited retinal dystrophies covering 104 genes. Panel-based testing for inherited retinal dystrophy genes was conducted by using the TruSight One Sequencing Panel kit on the Illumina NextSeq 500 platform (Illumina, San Diego, CA, USA).

Variations were annotated using Alissa Interpret Rev. 5.4.2 Agilent Inc. (SeqOne, Montpellier, France) by comparison with other databases (1000 Genomes Project, Exome Aggregation Consortium (ExAC), Ensembl, dbSNP, ClinVar, Human Gene Mutation Database (HGMD), Leiden Open Variation Database (LOVD), RetinoGenetics, RetNet, Mutation Database of Retina International) and predicted for pathogenicity with online bioinformatic tools (Sorting Intolerant From Tolerant (SIFT), PolyPhen, MutationTaster, MutationAssessor, and Ensembl Variant Effect Predictor). More precisely, to determine genomic variants of relevance, we selected putative disease-causing variants using the following criteria: variants previously reported as pathogenic; loss-of-function variants, such as stop–gain, frameshift, and small deletions or duplications (InDels); splice site variants; or missense variants predicted to be damaging or highly pathogenic. The variants were classified according to the current revised guidelines of the American College of Medical Genetics and Genomics (ACMG) [9]. 

Using this approach, we identified the homozygous missense mutation c.65T>C (p.Leu22Pro) in the RPE65 gene. The RPE65 gene is located on the 1p31 chromosome, contains 14 exons, and encodes a 65 kDa retinoid isomerase, which is expressed primarily in the retinal pigment epithelium and plays a vital role in the regeneration of 11-cis-retinol in the visual cycle [10]. The NM_000329.3(RPE65):c.65T>C (p.Leu22Pro) variant, a missense mutation causing the substitution of leucine with proline at position 22, has been shown to affect protein function in experimental studies [11]. This variant has been described in several studies on RPE65-related retinal dystrophies as a disease-causing mutation [12,13,14,15,16] and has been classified as pathogenic according to ClinVar (https://www.ncbi.nlm.nih.gov/clinvar/RCV000085218.21, accessed on 25 June 2024).

The identified variant was confirmed by Sanger sequencing and segregated in the family of the patient (daughter). No other family members of the patient were affected. The patient had a single unaffected daughter who was a healthy carrier of the same mutation.

We also tested a panel of genes associated with deafness by NGS, using the TruSight One Sequencing Panel kit (Illumina, San Diego, CA, USA), which gave a negative result.

**Figure 1:**
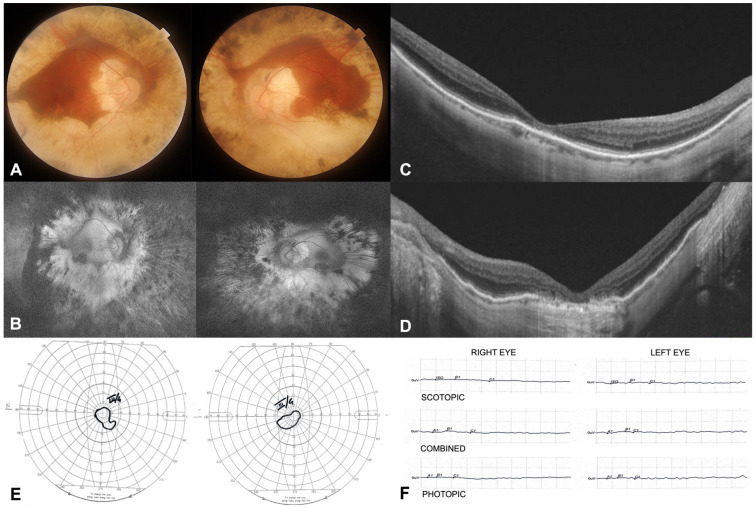
Multimodal imaging at baseline: (**A**) color fundus photography (Carl Zeiss Meditec, Jena, Germany) showing pale optic disc, attenuated retinal vessels, and bilateral and symmetrical areas of RPE atrophy distributed along the vascular arcades and extending temporally and nasally in a circular-shaped pattern; (**B**) ultra-wide field fundus autofluorescence (Daytona Optos; Marlborough, MA, USA) showing hyperautofluorescence of the sclera in the mid-pheriphery and posterior pole and hypoautofluorescence in the far peripheral retina; (**C**,**D**) spectral domain SD-OCT (Spectralis HRA+OCT device; Heidelberg Engineering, Heidelberg, Germany) of the RE (**C**) and LE (**D**) showing a thin fovea, and the ellipsoid band was impaired under the fovea in both eyes; (**E**) concentrically restricted Goldmann visual field in both eyes (III4e isopters); and (**F**) full-field standard electroretinography (Retimax; CSO, Florence, Italy) showing no recordable scotopic, photopic, or combined responses according to the ISCEV (International Society for Clinical Electrophysiology of Vision) Guidelines.

The patient’s best corrected visual acuity (BVCA) was 0.7 LogMar in the right eye (RE) and 0.92 LogMar in the left eye (LE). He was pseudophakic in both eyes. Intraocular pressure was within normal limits. Axial length was 29.60 mm in the RE and 30.28 mm in the LE. In both eyes, fundus examination showed waxy pallor of the optic nerve head with peripapillary atrophy, thin vessels, severe and diffuse retinal atrophy at the posterior pole circumscribing a central island of surviving retina, retinal pigment epithelium mottling and fine pigment clumping visible in the far periphery, and a posterior staphyloma (Figure 1A). A Goldmann manual visual field examination showed a concentrically restricted visual field of 10–20° in both eyes (Figure 1E). Fundus autofluorescence showed persistent hyperautofluorescence of the sclera in the mid-periphery and posterior pole and hypoautofluorescence in the far peripheral retina (Figure 1B).

Optical coherence tomography (OCT) examination showed a thin fovea with a central retinal thickness of 102 microns and 89 microns in the RE and LE, respectively (Figure 1C,D). A full-field electroretinography examination showed no recordable scotopic, photopic, or combined responses (Figure 1F).

Both eyes were treated with subretinal VN gene therapy; the surgeries were performed by an experienced vitreoretinal surgeon (F.G.). The patient received 1 mg/kg/day (up to 40 mg/day) of prednisone orally for 7 days, beginning 3 days before the first injection. Prednisone was tapered (0.5 mg/kg/day up to 20 mg/day) for the following 7 days or until 3 days before injection of the second eye, when the steroid regimen was repeated. A 25-gauge pars plana vitrectomy (CONSTELLATION Vision System; Alcon, Geneva, Switzerland) was performed under general anesthesia. Following standard core vitrectomy, posterior vitreous detachment was induced with preservative-free triamcinolone acetonide staining and an as complete as possible vitrectomy was then performed. The subretinal injection of VN was performed through a single retinotomy along the upper vascular arcades, avoiding vascular structures and areas of atrophy, 2 mm away from the center of the fovea, using a 41-gauge cannula (PolyTip Cannula 25 g/38 g; MedOne Surgical, Sarasota, FL, USA). The fovea was directly detached from the bleb formation in a single step; during the injection of VN, the injection pressure was manually controlled. Intraoperative OCT was acquired and showed a correct bleb formation process, without the detection of complications in both eyes. The patient received a single dose of 1.5 × 10^11^ vector genome VN in each eye, and each dose was planned to be delivered into the subretinal space at a total volume of 0.3 mL. Fluid–air exchange was then performed. Finally, sclerotomies were sutured with reabsorbable sutures (Vicryl 7/0). The Supplemental Digital Content demonstrates the surgical procedure (see Videos S1 and S2, which show the procedure for the right eye and left eye, respectively).

**Figure 2:**
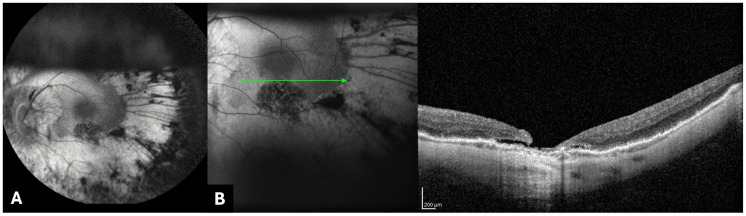
(**A**,**B**) Fundus autofluorescence (FAF) imaging and SD-OCT (Spectralis HRA+OCT device; Heidelberg Engineering, Heidelberg, Germany) at 1-week follow-up. (**A**) FAF imaging revealed a vitreous cavity half-filled with air. (**B**) OCT examination showed a partial-thickness macular hole, inferiorly to the fovea.

**Figure 3:**
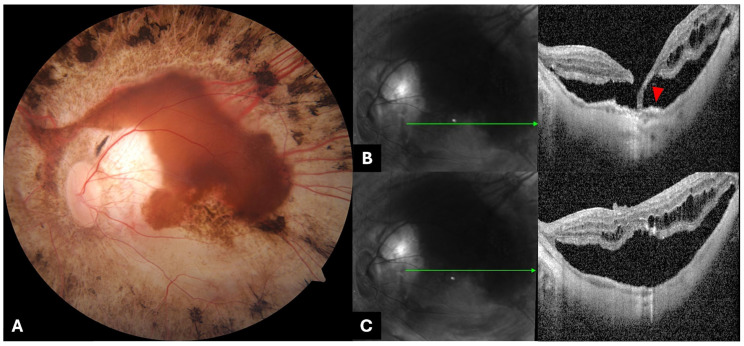
(**A**) Color fundus photography (Carl Zeiss Meditec, Jena, Germany) and (**B**) spectral domain OCT scans (Spectralis HRA+OCT device; Heidelberg Engineering, Heidelberg, Germany) of the LE at 3-week follow-up showing retinal detachment at the posterior pole due to a full-thickness macular hole. The retinal detachment was located at the posterior pole and reached the areas of RPE atrophy in the mid-periphery. (**B**) SD-OCT showed a full-thickness macular hole. The temporal edge of the hole was partially attached by a bridge of thin retinal tissue (red arrowhead) to the RPE, while the nasale edge was smooth and elevated. (**B**,**C**) Irregularities of the photoreceptor outer segment were visible at the macular hole borders. Some retinal cysts were also detectable in the inner retina layers.

The RE was the first eye treated, and the post-operative course was good, without complications. The LE was treated two weeks later with the same technique, but, at the 1-week post-operative follow-up, we noticed the presence of a partial thickness macular hole, inferiorly to the fovea (Figure 2), and, at the 3-week follow-up, macular detachment occurred. Fundus examination and OCT of the LE clearly revealed retinal detachment at the posterior pole due to a full-thickness macular hole (Figure 3).

The patient complained of loss of vision in the LE, with BCVA in the LE reduced to light perception. The macular detachment was managed with a 23-gauge pars plana vitrectomy, 0.18% Trypan Blue and 0.03% Blulife membrane staining, inverted internal limiting membrane (ILM) flap peeling, and sulfur hexafluoride (SF6) 20% tamponade. We observed hyaloid remnants at the posterior pole and barely adherent membranes, requiring high tractional force during the peeling (see Supplementary Video S3).

**Figure 4:**
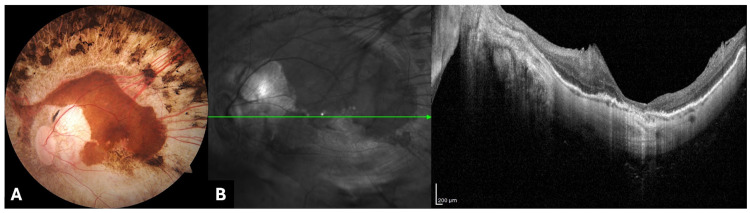
(**A**) Color fundus photography (Carl Zeiss Meditec, Jena, Germany) and (**B**) optical coherence tomography (Spectralis HRA+OCT device; Heidelberg Engineering, Heidelberg, Germany) 40 days after the second surgery on the LE. The retina was completely reattached and a post-surgery OCT scan showed macular hole closure.

Four days after the second surgery, the gas was completely reabsorbed, the macular hole was closed, and the macular detachment was resolved. The BCVA improved to the baseline value, 0.92 Logmar. Figure 4 shows the fundus appearance and OCT scan of the LE at 40-day follow-up after the second surgery.

**Figure 5:**
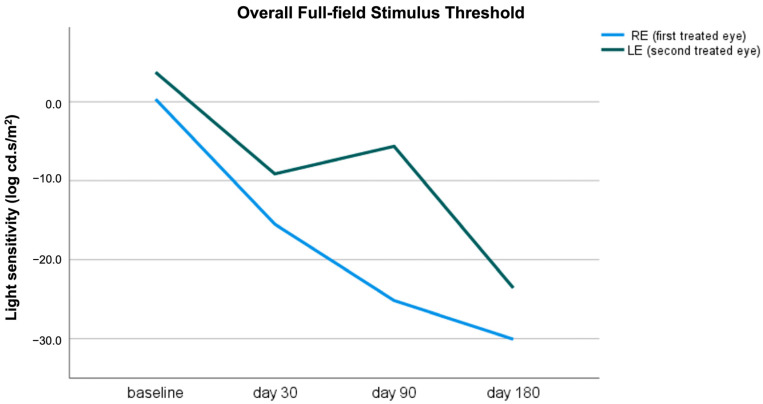
Overall full-field stimulus threshold outcomes in the right eye (RE) (blue line) and left eye (LE) (green line). A significant improvement (i.e., >10 dB) in the light sensitivity threshold was clearly detectable on day 30 and during the follow-up.

**Figure 6:**
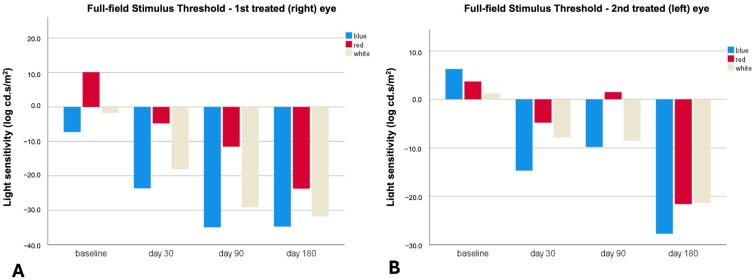
Full-field stimulus threshold outcomes in the right eye (**A**) and left eye (**B**). A significant improvement in the light sensitivity threshold in response to all three stimulus colors was observed in both eyes at the earliest post-treatment time point. This improvement was also evident after the second surgery (day 30) for the left eye and at the end of the follow-up (day 180).

The effect of treatment on visual function was assessed using a full-field stimulus threshold (FST), conducted using the Espion E3 Color Dome (Diagnosys LLC, Lowell, MA, USA). We report the FST measurements for the two treated eyes and the FST values for the three stimulus thresholds (blue, red, and white) at baseline, after subretinal gene therapy, and at the end of follow-up (Table 1 and Table 2 and Figure 5 and Figure 6). Notably, an improvement in FST values was evident in both eyes after subretinal injection at the earliest post-treatment time point (day 30), even in the LE, which underwent a second surgery due to a macular hole and retinal detachment 21 days after the subretinal gene therapy. The improvement in light sensitivity measured using FST was clearly evident at the end of the follow-up (day 180) in both treated eyes. This peculiar aspect confirmed the efficacy of the gene therapy and suggested a rapid improvement of cone and rod function, despite complications that may have negatively influenced the treatment effectiveness, as seen in the LE of our patient.

## 3. Discussion and Conclusions

We report a case of a patient with RPE65 retinal dystrophy exhibiting an uncommon phenotype who developed a macular hole and detachment following the subretinal injection of VN. We also describe the successful surgical management of this complication.

Notably, the retinal phenotype expression of the RPE65 mutation was not typical in this patient, but it was characterized by a severe and diffuse retinal atrophy circumscribing a central island of surviving retina at the posterior pole, very high axial length, and posterior staphyloma. While many patients with RPE65-associated retinal dystrophy usually present a widespread loss of autofluorescence, our patient showed marked hypoautofluorescence in the far periphery, but it was associated with the persistence of retinal autofluorescence in the central retinal region. Moreover, the whole phenotype of this patient was uncommon in comparison to the most frequent clinical characteristics found in RPE65-associated retinopathy [14,17]. The patient showed an early onset, a progressive loss of visual acuity and visual field, and an abnormal electroretinographic response, which are typical features of RPE65-associated retinopathy; however, he had an uncommonly slow progression of the disease, with a relatively maintained visual acuity at 70 years of age, different from the typical expression of RPE65-associated retinopathy, which is characterized by a more severe course, with legal blindness occurring before the age of 40 years old. Less aggressive forms of RPE65-associated retinopathy with slow progression are reported in the literature, as described by Merle et al. [18], which may qualify for subretinal gene therapy with VN.

The patient described in our study was affected by severe bilateral hearing loss, needing hearing aids since he was 50 years old. To rule out genetic causative mutations of hearing loss, we performed adjunctive genetic tests that did not identify any other mutations.

The patient underwent VN administration in both eyes, but, after treatment, he developed in his most myopic eye (LE) a macular hole and detachment, two complications rarely reported after VN subretinal injection [19,20].

In the 3- and 4-year studies of a phase III trial [21], only one patient developed a retinal detachment. The retinal detachment occurred 4 years after the VN administration. The causative mechanism of retinal detachment was related to the presence of proliferative retinopathy caused by the administration procedure and not the drug itself. In fact, no causative retinal breaks or reopening of the retinotomy could be detected in those patients at fundus examination. On the other hand, macular “blow out” with hole formation during subretinal injections of different drugs is a well-recognized complication and may depend on the speed and volume of the injection [20,22,23,24]. This complication has been rarely reported during the subretinal injection of gene therapies [25].

Currently, no widely accepted technical approach exists for the subretinal delivery of AAV gene therapy in RPE65 patients. There are some concerns regarding foveal detachment during a subretinal injection procedure: some physicians recommend directly detaching the fovea during the injection of VN with a single-step bleb formation, while others advise against the detachment of the foveal region due to the unpredictable behavior of the fragile retinal tissue, with thin overlying neuroretina and progressive RPE cell degeneration. Some surgeons recommend using a balanced salt solution to separate the retina from the RPE before re-entering with drug delivery through the same retinotomy in a two-step approach [26,27,28]. Furthermore, some physicians suggest performing subretinal injections using a foot-pedal control system that allows closer pressure monitoring readings during gene therapy delivery and the setting of a maximum injection pressure limit at a level that creates a continuous flow [29]. This degree of control and prevention in fact is not feasible when performing subretinal injections manually.

In our case, we cannot report drug delivery rate and injection pressure data, as it was manually controlled. The fovea was directly detached with a single-step bleb formation during the injection of VN through a single retinotomy. The technique was the same in both eyes, and intraoperative OCT showed a correct bleb formation process, without the detection of complications, but the macular hole and detachment occurred only in the LE.

We hypothesized that in this case, the high axial length and posterior staphyloma may represent adjunctive risk factors in the hole formation. Moreover, the preoperative OCT scan showed a thinner fovea in the LE than in the RE, especially in the macular area inferior to the fovea, adjacent to the inferior atrophy, at the same point where the macular hole developed. We hypothesized that this extremely thin macular zone, near the atrophy, was the weak point that opened after the fluid injection into the subretinal space. This hypothesis may be further corroborated by the fact that 7 days after VN administration, subretinal fluid was almost reabsorbed and a partial splitting of the inner retinal layer was already detectable upon OCT examination. More precisely, OCT examination showed that the nasal edge of the hole was elevated and already detached from the RPE, whereas the temporal side was partially attached by a thin bridge of tissue to the RPE layer. This resulted in a full-thickness macular hole with subsequent retinal detachment 14 days later. Moreover, during the second surgery, we observed hyaloid remnants at the posterior pole and barely adherent macular membranes requiring high traction force during peeling. We hypothesized that these findings were indirect signs of vitreoschisis and incomplete hyaloid detachment during the first surgery.

Fortunately, the macular detachment was surgically treated without sequelae. The patient underwent a 23-gauge pars plana vitrectomy with ILM peeling and inverted ILM flap technique with SF6 20% tamponade. Currently, the inverted ILM flap peeling procedure has a primary role in the surgical repair of large or myopic macular holes [30,31] and was effective also in our case.

Moreover, we may speculate that the inverted flap technique had an adjunctive beneficial effect on our patient. We suppose that the ILM, filling the macular hole of this patient, may have acted as a scaffold for the activation and migration of Muller cells and may have strengthened the fragile macular tissue of this RPE65 patient, contributing to the closure of the macular hole.

Six months after the second surgery, the macular hole was still closed, the retina was attached, BCVA was maintained, and the full-field stimulus threshold test improved, similar to the fellow eye.

We speculate that in eyes with atypical phenotypes, as in our case, characterized by retinal thinning, high myopia, posterior staphyloma, and extensive atrophy circumscribing the macula, the volume of drug injected may be adjusted, and controlled injection pressure may reduce the risk of macular hole formation. It is also essential to ensure that the hyaloid detachment is complete before the subretinal injection. Moreover, we suggest that patients with atypical phenotypes be informed that the risk of subretinal VN injection may be higher than for patients with typical phenotypes. We believe that it is essential to preoperatively identify areas of excessive retinal thinning, which could open under subretinal pressure to form a secondary retinal hole due to the retinal stretching during the bleb propagation.

In conclusion, our experience suggests that patients with atypical RPE65 phenotype expression and high myopia who undergo VN subretinal injection must be carefully examined preoperatively, intraoperatively, and postoperatively. This approach is essential to reduce the risk of macular hole and detachment and promptly detect and manage these potential complications.

## Figures and Tables

**Table 1:** Overall full-field stimulus threshold outcomes of the treated eyes. Values are expressed as means ± standard deviations.

Overall FST (log cd.s/m^2^)				
	Baseline	Day 30	Day 90	Day 180
RE	0.3 ± 8.8	−15.5 ± −9.6	−25.1 ± −12.1	−30.1 ± −5.7
LE	3.7 ± 2.5	−9.1 ± −5.0	−5.6 ± −6.2	−23.5 ± −3.5

FST: full-field stimulus threshold; RE: right eye; LE: left eye.

**Table 2:** Full-field stimulus threshold white, blue, and red measurements in the right eye and left eye during follow-up.

		Baseline		Day 30		Day 90		Day 180	
		RE	LE	RE	LE	RE	LE	RE	LE
FST (log cd.s/m^2^)	white stimulus	−1.8	1.2	−18.1	−7.9	−29.1	−8.6	−31.8	−21.4
	blue stimulus	−7.3	6.3	−23.6	−14.7	−34.9	−9.8	−34.7	−27.7
	red stimulus	10.1	3.7	−4.8	−4.8	−11.5	1.5	−23.7	−21.6

FST: full-field stimulus threshold; RE: right eye; LE: left eye.

## Data Availability

Data is contained within the article or Supplementary Material.

## Supplementary Materials

The following supporting information can be downloaded at: https://www.mdpi.com/article/10.3390/genes15070879/s1.
